# The Role of Androgen Receptor Mutations in Prostate Cancer Progression

**DOI:** 10.2174/138920209787581307

**Published:** 2009-03

**Authors:** G.N Brooke, C.L Bevan

**Affiliations:** Androgen Signalling Laboratory, Department of Oncology, Imperial College London, London, W12 0NN, UK

## Abstract

Prostate tumour growth is almost always dependent upon the androgen receptor pathway and hence therapies aimed at blocking this signalling axis are useful tools in the management of this disease. Unfortunately such therapies invariably fail; and the tumour progresses to an “androgen-independent” stage. In such cases androgen receptor expression is almost always maintained and much evidence exists to suggest that it may still be driving growth. One mechanism by which the receptor is thought to remain active is mutation. This review summarises the present data on androgen receptor mutations in prostate cancer, and how such substitutions offer a growth advantage by affecting cofactor interactions or by reducing ligand specificity. Such alterations appear to have a subsequent effect upon gene expression suggesting that tumours may “behave” differently dependent upon the ligand promoting growth and if a mutation is present.

## 1 THE ANDROGEN RECEPTOR

The Nuclear Receptor family is the largest group of eukaryotic transcription factors, with 48 human receptors presently described [1]. The androgen receptor (AR) is a steroid receptor; a sub-family of receptors characterised as ligand dependent, sequence specific transcription factors [2] and like other nuclear receptors the AR has a modular structure (reviewed in [1]) - see Fig. (**1**). The AR gene is situated at Xq11-12 and consists of 8 exons with exon 1 encoding the N-terminal domain and the entire 5’ untranslated region, exons 2 and 3 encoding the DNA binding domain (DBD) and exons 4-8 encoding the “hinge” region and ligand binding domain (LBD) [3].

### 1.1 Activation Function 1

The N-terminal domain contains activation function-1 (AF-1), which is composed of 2, to some extent separable, transcription activation units (TAUs) – TAU1 and TAU5 (residues 1-485 and 360-528 respectively) [3]. Although the locations of the TAUs overlap, the cores of these units (containing over 80% activity) are in separate regions and have individual characteristics. TAU1 appears to be important in ligand-dependent activation, whereas deletion of the LBD reduces the activity of TAU1; instead, most activity is *via* TAU5 [4].

A polymorphic polyglutamine (CAG) tract is present in the N-terminus of the AR and ranges from 8 to 30 repeats in normal individuals, with a modal length of 20 [5]. The rare neuromuscular disorder, spinal and bulbar muscular atrophy (SBMA or Kennedy’s disease), is caused by expansion of the CAG tract to more than 40 repeats. Receptors with this expansion form nuclear aggregates and cause neurodegredation through a toxic gain-of-function mechanism [6, 7]. The mechanism of this toxicity is not clear, but it has been postulated that abnormal expression of caspases, in response to receptor aggregates, may be involved (reviewed in [7]). Longer polyglutamine tract length also results in decreased AR transcriptional activity *in vitro* [8], whereas shorter CAG repeats has been linked to increased activity and subsequent increased risk of prostate cancer [9].

The N-terminal region of the AR appears to be highly unstructured. A more folded and subsequently proteosome resistant conformation can be achieved by incubation with folding-inducing solvents (such as trimethylamine-N oxide, TMAO), and also by binding of the cofactor TFIIF (Transcription Factor IIF) [10, 11]. This increased folding is important in transcriptional activity since it enhances recruitment of accessory proteins such as SRC1 (Steroid Receptor Coactivator 1) [12].

### 1.2 DNA Binding Domain

The DNA binding domains (DBD) of nuclear receptors have high sequence homology, with differences in the amino acids that contact the DNA eliciting specificity [13]. The DBD contains 9 cysteine residues, 8 of which form 2 tetrahedral conformations each around a single zinc atom, forming 2 zinc finger-like modules through which the receptors interact with DNA [14]. The first zinc finger contains the P-box (Gly^577^, Ser^578^ Cys^579^, Lys^580^ and Val^581^ of the AR), which from studies using hybrid receptors has been found to confer receptor response element specificity [13, 15-17]. The second zinc finger appears to stabilise binding *via* interaction of the D-box (amino acids Ala^596^, Ser^597^, Lys^598^, Asn^599^ and Asp^600^) with the sugar-phosphate backbone of DNA [17].

### 1.3 Hinge

The hinge region of nuclear receptors was originally thought to be a flexible linker region important in DNA binding and dimerisation. Detailed analysis of the region, however, has demonstrated that the region plays a more diverse role than initially thought [18, 19]. Using deletion mutants, Haelens *et al*. found that amino acids 629-636 of the hinge region are also important in nuclear localisation, protein interactions and the ligand dependent N-/C-terminal interaction [19].

### 1.4 Ligand Binding Domain/Activation Function 2

The ligand binding domain of nuclear receptors is comprised of 11-12 α-helices and 1 β-sheet that fold to form a triple-layered anti-parallel α-helical sandwich. This folding forms a hydrophobic pocket into which the ligand fits; crystallographic analysis of various members of the family have revealed a highly conserved structure with the exact dimensions of the pocket varying according to the cognate ligand [20-30]. Ligand binding promotes the relocalisation of helix 12, which realigns to create a surface consisting of helices 3, 4, 5 and 12 that is important in cofactor binding [31-33].

The predominant activation function in the AR appears to be AF-1, since deletion of AF-2 and the LBD results in a constitutively active receptor with similar activity to the wild-type receptor in the presence of agonist [34, 35]. Recently, however, it has been demonstrated that AF-2 plays a significant role in chromatin and hence its importance in transcriptional regulation may have been underestimated [36].

### 1.5 The Androgen Receptor Pathway

The unliganded AR is predominantly cytoplasmic [37, 38] and associated with a large heterocomplex, including chaperone and heat-shock proteins, that holds the receptor in a ligand binding competent state [39] (Fig. **2**). Ligand binding promotes dissociation from this complex, dimerisation, nuclear localisation and intra- and inter-receptor interactions [40]. The AR, like several other nuclear receptors (e.g. oestrogen receptor α and the progesterone receptor) undergoes a ligand-dependent N- and C-terminal interaction. This interaction is predominantly mediated by an N-terminal ^23^FQNLF^27^ motif interacting with the AF-2 cofactor-interaction surface. It is important for transcriptional activity since it increases receptor stability, reduces ligand off-rate and creates interaction sites for accessory proteins [41, 42].

The AR has been found to interact with a large number of proteins that enhance receptor activity, termed coactivators. Such factors often have, or recruit proteins that have, histone acetyltransferase activity and are hence believed to increase receptor activity in part by relaxing chromatin structure. The best characterised are the p160 family of coactivators, consisting of SRC-1/NCOA1 (Steroid Receptor Coactivator-1 / Nuclear Receptor Coactivator 1), SRC-2/NCOA2/TIF-2/GRIP-1 (Transcriptional Intermediary Factor 2 / Glucocorticoid Receptor Interacting Protein-1) and SRC-3/NCOA3/AIB1/pCIP/RAC-3 (Amplified In Breast Cancer-1 Protein / CBP-Interacting Protein / Receptor-Associated Coactivator 3) [43]. Many coactivators interact with nuclear receptors *via* LxxLL motifs (where L is leucine and x is any amino acid), which form an amphipathic α-helix that binds directly to the hydrophobic AF-2 coactivator groove [44, 45]. Unlike the other steroid receptors, the AR can also interact with and has higher affinity for phenylalanine rich motifs, for example the FQNLF motif found in the N-terminus of the AR and those found in some coactivators, for example Androgen Receptor Activator 70 (ARA70) [46, 47]. Study of the crystal structure of the AR AF-2 surface has demonstrated the coactivator groove to be much deeper than that found in the other steroid receptors [47, 48]. This deeper groove appears to be able to accommodate the bulkier phenylalanine residues hence explaining the differences in receptor-interaction motif specificity.

Conversely, corepressor proteins have also been identified, for example Nuclear Co-Repressor Protein (NCoR) and Silencing Mediator for Retinoid and Thyroid Hormone Receptor (SMRT), which have been found to bind to both agonist- and antagonist-bound receptors and reduce their activity. There are multiple mechanisms by which corepressors appear to inhibit receptor signalling - for example, *via* recruitment of histone deacetylases to condense chromatin structure, and *via* nuclear exclusion of the receptor [49].

The AR interacts with DNA *via* response elements located within the regulatory regions of target genes. The AR has been found to bind strongly to an inverted repeat of a 5’-TGTTCT-3’ half site (termed the core recognition sequence) separated by 3 base pairs [50-52]. This consensus sequence is not specific for the AR, but also acts as a response element for glucocorticoid, mineralocorticoid and progesterone receptors [51, 53, 54]. Recently a second class of response elements, which appear to be highly AR specific, have been described and these consist of a direct repeat of the core recognition sequence. Both inverted and direct repeats have been found in the regulatory regions of many androgen responsive genes, such as *PSA* (Prostate Specific Antigen) and *SC* (Secretory Component) [55-58].

## 2 THE PROSTATE

The prostate is a secretory gland located at the base of the bladder with a composition that is approximately 70% glandular elements (acini that empty into multiple small ductules) and 30% fibromuscular stroma [59]. The stroma is continuous with the capsule that encases the prostate, consisting of collagen, elastin and smooth muscle. The muscle contracts upon ejaculation, forcing prostatic secretions, important in events such as semen coagulation and liquefaction, into the urethra [60]. The first link between androgens and prostate development was made by John Hunter, who in 1786 noted that the size of the gland in castrated animals was significantly reduced compared to that in intact animals [61]. The prostate has since been demonstrated to develop from the urogenital sinus in response to fetal testicular androgens (reviewed in [62]). At maturity growth of the gland ceases, but androgens continue to play an important role in prostate function. In some men, androgen dependent growth of the prostate resumes, resulting in benign prostatic hyperplasia (BPH), premalignant prostatic intraepithelial neoplasia (PIN) or prostate cancer (PCa).

### 2.1 Prostate Cancer

Prostate cancer is the most common cancer in men in the United Kingdom, with approximately 35,000 men diagnosed every year [63]. The biggest risk factor in prostate cancer is age, with more than 60% of cases occurring in men over 75. Incidence of prostate cancer have been consistently rising and hence it is likely that prostate cancer will overtake lung cancer as the leading cause of cancer related death in Western men.

### 2.2 Treatment

Approximately 25% of prostate cancer patients have organ-confined disease upon presentation and for such patients radical prostatectomy (complete removal of the prostate) offers the highest likelihood of long-term disease free survival [64]. Unfortunately the majority of patients present with disease that has spread from the prostate capsule and hence surgery is not an option. Since the growth of the prostate is almost always dependent upon the AR pathway, therapies to treat non-organ confined disease often target this signalling axis. Up to the 1950s orchiectomy was routinely used to reduce circulating levels of androgens, since the main site of production is the testis. Since then chemical castration has been the preferred method and this is achieved by using leuteinising hormone releasing hormone (LHRH) analogues, which act *via* the pituitary-hypothalamus signalling axis to block androgen production. These analogues successfully reduce circulating levels of testosterone by more than 95%, but levels of adrenally produced androgen precursors such as dehydroepiandrosterone remain unaffected, and these can be effectively converted in the prostate into the potent androgen dihydrotestosterone [65, 66]. Hence androgen levels within the prostate may only be reduced by approximately 60% and for this reason antiandrogens are often also administered to reach “total androgen blockade”.

Antiandrogens are ligands that can bind to the AR and hold it in an inactive state. The exact mechanisms of antiandrogen action are not completely understood, but they appear to function at least in part *via* the recruitment of corepressors to the regulatory regions of target genes. Shang *et al*. for example, found that the corepressors NCoR and SMRT were recruited to the promoter region of the *PSA* gene following treatment with the antiandrogen Bicalutamide [67]. In contrast, coactivators such as SRC1 were present following treatment with an agonist.

### 2.3 Androgen Independence

Hormone therapy is successful in the majority of patients, resulting in both symptomatic and pathological improvement. Unfortunately this therapy invariably fails after a median of 2 years and the tumour progresses to a more aggressive “androgen-independent” stage. To call this stage androgen- or hormone- independent is perhaps misleading since in most cases the AR is expressed and much data suggests that the receptor is still functional. Several mechanisms have been proposed to explain how the AR may still be driving growth even in the androgen-depleted environment and these include AR amplification, alterations in cofactor levels and AR mutation [68].

### 2.4 AR mutations

In early stages of prostate cancer mutations of the AR are rare but their frequency is significantly increased in advanced, androgen-independent tumours suggesting that AR mutations play a role in tumour progression [69-72]. Marcelli *et al.* for example, found that out of 99 patients in the early stages of prostate cancer, none had mutation(s) in the AR coding sequence [70]. In advanced stages of the disease, however, 8 out of the 38 patients studied (21%) with more advanced disease, were found to have mutation(s) of the AR.

Over 70 different somatic missense AR mutations have been described in patients with prostate cancer [73]. Shi *et al*. compared the activity of 44 such mutations and found that 20 had a gain of function [74]. Cells carrying such mutations are likely to provide a growth advantage in the androgen-depleted environment and hence be selected for during therapy. The mechanisms by which such mutations provide a growth advantage appear to be, at least in part, due to alterations in cofactor recruitment or by reducing ligand specificity.

#### 2.4.1 Alterations in Cofactor Binding

**Fig. (1):**
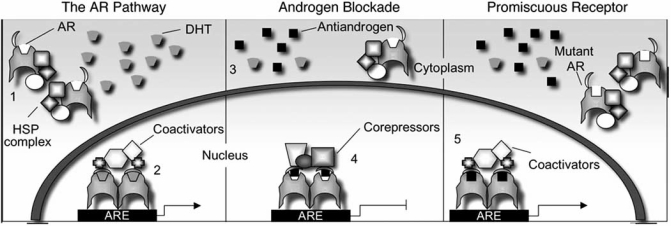
**Frequency and location of androgen receptor mutations associated with prostate cancer.** The location and frequency of different single base mutations are given, highlighting where these substitutions lie in relation to functional domains of the androgen receptor.

**Fig. (2):**
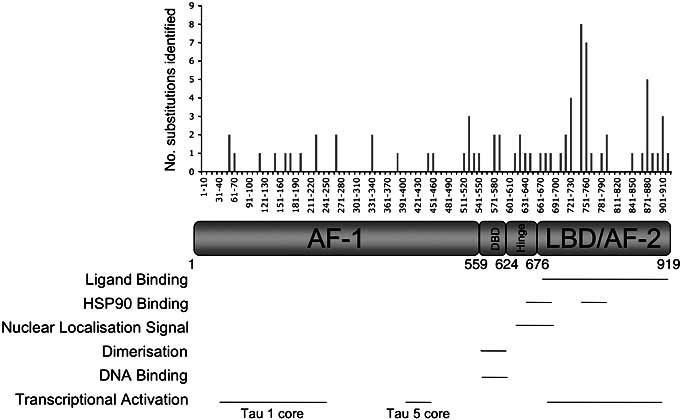
**Hormone therapy failure caused by AR mutation.** (**1**) The unliganded AR exists in the cytoplasm associated with a large heterocomplex that holds it in a ligand binding competent state. (**2**) Upon ligand binding the receptor dimerises, translocates to the nucleus where it binds DNA and promotes gene transcription *via* the recruitment of accessory proteins, such as coactivators. (**3**) Non organ-confined prostate cancer is usually treated by blocking the AR pathway. This is achieved by blocking the production of androgen (using LHRH analogues) and/or antiandrogens. (**4**) Antiandrogens bind to the AR but do not promote an active conformation and instead block receptor function, at least in part, by promoting the recruitment of corepressors to the regulatory regions of target genes. (**5**) This therapy selects for cells that have mechanisms by which the tumour can grow in the androgen depleted environment. One such mechanism is that of AR mutation and in some cases these mutants provide a growth advantage because they reduce the ligand specificity of the receptor. Hence other ligands, such as the antiandrogens being used in treatment, are now able to promote an active conformation, the recruitment of coactivators and subsequently gene expression.

Although most mutations lie in the LBD, more than 30 substitutions associated with PCa have been identified in other parts of the receptor (Fig. **1**). Tilley *et al*. for example, identified dual somatic missense mutations within the N-terminal polyglutamine tract [75], which resulted in interruption of the tract by two leucine residues. These substitutions were found to reduce the ligand induced N- and C-terminal interaction, but paradoxically led to a receptor with greater activity than the wild-type AR. Coactivators have been described that interact with the CAG repeat, for example ARA24 (Androgen Receptor-Associated Protein 24) [76], and it is believed that these mutations confer greater transcriptional activity due to increased stability and folding of the tract, enhancing such interactions. This was demonstrated by the finding that ARA24 was found to enhance activity of the mutant to a greater extent than the wild-type receptor [75]. It is possible that these enhanced interactions with coactivators may potentiate AR signalling in low levels of androgen or in the presence of weaker agonists and thus contribute to therapy failure.

Mutations have also been identified which disrupt an inhibitory domain located within the AR. The activity of AF-2 has been demonstrated to be inhibited by the hinge region [77]. Haelens *et al*. have studied the effect of two mutations, R629Q and K630T, located in the hinge region of the AR. These mutations, which lie in the bipartite nuclear localisation signal, reduced nuclear localisation and DNA binding of the receptor. Surprisingly, however, the mutants had greater transcriptional activity than the wild-type receptor. Similarly Buchanan *et al.* found that mutations Gln668Arg and Ile670Thr, also within the hinge region, also had increased activity and reduced ligand specificity compared to the wild-type receptor without changes in receptor levels, ligand binding or DNA binding [78]. The data therefore suggests that mutations of the hinge region provide a growth advantage by increasing AR activity through disruption of an inhibitory domain. The exact mechanisms by which the hinge region inhibits the activity of AF-2 is yet to be elucidated, however, it has been postulated that the region may be inhibiting coactivator interactions [19]. Hence, mutations of the hinge region may increase receptor activity by attenuating coactivator recruitment.

#### 2.4.2 Alterations in Ligand Specificity

The first AR variant with loss of ligand specificity to be described was a threonine to alanine substitution at amino acid 877 [79]. This mutant has since been frequently found in advanced prostatic carcinomas - Taplin *et al.* for example, found the mutation in 30% of bone marrow metastases [69]. The receptor not only responds to androgens but is also activated by oestrogens, progestins and the antiandrogens cyproterone acetate and hydroxyflutamide (the active form of flutamide) [80]. Crystal structure analysis of the AR LBD has revealed that threonine 877 forms hydrogen bonds with the 17β-hydroxyl group of androgen [30]. Further modelling has demonstrated that substitutions to the smaller alanine affects the size and shape of the receptor such that other ligands can fit into the pocket and induce an active conformation [81].

Not all prostate cancer-associated substitutions in the LBD reduce ligand specificity by altering the dimensions of the pocket. The H874Y mutant AR, for example, is also activated by hydroxyflutamide, oestradiol, progesterone, and cyproterone acetate, but the side chain of this residue points away from the pocket and is buried in a cavity between helices 11 and 12, which is formed following ligand induced activation [82]. This cavity is large enough to accommodate the tyrosine aromatic ring and hence it appears unlikely that the altered ligand specificity is as a result of steric alterations at this site. Instead, it appears that the more hydrophobic tyrosine side chain strengthens the interaction of helix 12 with this groove. It has been proposed that this stronger interaction could promote the relocation of helix 12 to the active position even in the presence of a ligand that does not optimally fit into the pocket, thus reducing ligand specificity [82]. Interestingly, the mutant has been found to have enhanced binding to the p160 coactivators, suggesting that, in addition to broadened ligand specificity, the mutant receptor also has enhanced coactivator recruitment [83, 84].

Differences in coactivator binding have also been found dependent upon which ‘agonist’ is activating the mutant receptor. As described previously, the AR can bind both LxxLL and phenylalanine-rich motifs (such as FxxLY), but has higher affinity for the latter. We have studied the preference of several of the most commonly identified mutant receptors (H874Y, T877A and T877S) for these motifs and found striking differences in motif utilisation dependent upon which ligand is activating the receptor [85]. In the presence of cyproterone acetate, for example, the mutants specifically interact with the LxxLL motif whereas in the presence of hydroxyflutamide the receptor interacts with the FxxLY motif. Using chromatin immunoprecipitation, siRNA and target gene expression analysis, we were able to show that this selectivity extended to coactivator recruitment to endogenous genes demonstrating that dependent upon ligand and interaction motif, the mutant receptors may utilise different subsets of coactivators to potentiate gene expression.

**Fig. (3):**
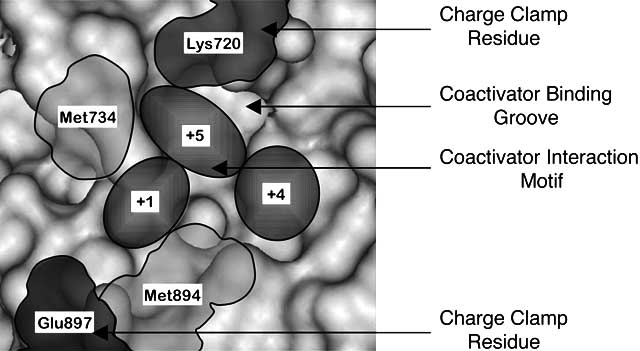
**Surface representation of the AR coactivator groove.** Representation of the AR AF-2 surface highlighting the L-shaped cleft and key residues important in coactivator binding. +1, +4 and +5 refer to the regions in which the 1^st^, 4^th^ and 5^th^ amino acids of LxxLL and FxxLF-like motifs lie following binding. Image created using RasMol V2.6 using co-ordinates from [30].

The coactivator interaction groove of the AR is comprised of an L-shaped cleft comprised of three distinct subsites (formed from helices 3, 4, 5 and 12 of the LBD) that bind hydrophobic groups at the +1, +4 and +5 positions in cognate peptides [47]. The conserved charge residues at either end of the cleft, Lys702 and Glu897, form what is referred to as the “charge clamp” (Fig. **3**). The charge clamp residues form electrostatic interactions with the main chain atoms at either end of phenylalanine-rich motifs whereas LxxLL motifs only form hydrogen bonds with Lys720. Charge clamp residue Glu897 is located in helix 12, the positioning of which is likely to be affected by the agonist bound. Therefore if an agonist is bound that does not induce the correct positioning of the charge clamp residues for interaction with phenylalanine rich motifs, then the site appears to be available for LxxLL motif binding.

Several studies have demonstrated that inter- and intra-receptor interactions made by the AR have differential effects dependent upon promoter context [86-88]. We hypothesised that a mutant receptor activated by different agonists could therefore regulate different subsets of genes, hence we studied the expression levels of genes involved in prostate differentiation (*Kallikrein 2, KLK2,* and *Differentiation Regulated Gene-1, DRG-1*) and cell cycle progression (*Cyclin Dependent Kinases 2 and 4, CDK2 and CDK4*) in the LNCaP prostate cancer cell line, which endogenously expresses the T877A mutant AR [85]. The expression of *KLK2* and *CDK2* in response to different ligands was similar – induced most strongly by androgen, then the antiandrogen hydroxyflutamide then cyproterone acetate. Evidence of *CDK4* being an androgen-regulated gene is contradictory, with upregulation in response to androgen reported in some studies [89] but not found in others [90]. In agreement with the latter we found no induction of *CDK4* in response to androgen, but interestingly the two antiandrogens did induce expression. Even more striking was the regulation of *DRG-1,* which we found to be highly upregulated by androgen (more than 12-fold) and only weakly by hydroxyflutamide (approximately 1.7-fold). Hence the mutant AR induced different “patterns” of regulation of a subset of androgen-regulated genes according to the ligand. Since this includes genes that are involved in tumour growth, there may be implications for tumour progression and treatment.

## 3 IMPLICATIONS FOR THE TREATMENT OF PROSTATE CANCER

The findings summarised here have important implications for the treatment of prostate cancer. The data suggest that tumours may behave differently dependent upon (i) which mutation, if any, is present and (ii) which ligand is driving growth, since different subsets of genes may be regulated. We suggest that it would therefore be useful to screen patients for AR mutations following hormone therapy failure, so that subsequent treatment could be adjusted accordingly. Understanding of how AR mutations alter androgen signalling at the molecular level will also be useful in the development of novel therapies, in particular Selective Androgen Receptor Modulators (SARMs). Knowledge of how such molecules affect the structure of the AR, interactions that the receptor makes and subsequently gene expression could aid in designing drugs that regulate certain subsets of genes. For example, it would be desirable to design a SARM that blocks expression of androgen target genes that promote tumour growth, whilst up-regulating transcription of beneficial genes (for example those important for maintenance of bone density).
